# Case Report: Dual impact of daratumumab in early T-cell precursor acute lymphoblastic leukemia -- consolidation therapy achieves remission and eliminates donor-specific HLA antibodies

**DOI:** 10.3389/fimmu.2026.1734705

**Published:** 2026-01-22

**Authors:** Kye Ling Wong, Tertius Tuy, Jeffrey Quek, Yeh Ching Linn

**Affiliations:** Department of Haematology, Singapore General Hospital, Singapore, Singapore

**Keywords:** acute lymphoblastic leukemia, daratumumab, desensitization, donor-specific anti HLA antibodies, hematopoietic stem cell transplant

## Abstract

Desensitization of antibodies against human leucocyte antigen (HLA) is an important step in allogeneic hematopoietic stem cell transplantation with mismatched or haploidentical donors. Various strategies have been established to reduce donor specific HLA antibody (DSA) and reduce risk of graft failure. These strategies target a few pathways including depleting B cells, altering plasma cells, removing as well as modifying antibodies. We present a case whereby use of the anti-CD38 monoclonal antibody daratumumab was able to successfully reduce DSA via its action of the cell surface glycoprotein highly expressed on plasma cells in a multiparous patient with early T-cell precursor acute lymphoblastic leukemia (ETP-ALL). In this case, use of daratumumab was originally intended for consolidation therapy of ETP-ALL as treatment related complications encountered during induction therapy precluded use of further systemic chemotherapy. Choice of the anti-CD38 monoclonal antibody was supported by pre-clinical studies suggesting that CD38 is robustly expressed in T-ALL blasts thereby making it a potential effective target for daratumumab. It was through the inadvertent use of this novel agent for ETP-ALL consolidation therapy that a coinciding benefit of reduction in DSA was also achieved. In summary, daratumumab can be considered as a second line or salvage option for desensitization of DSA prior to haploidentical stem cell transplantation especially in multiparous ethnic-minority patients whom we often grapple with the issues of lack of donor availability and multiple DSA.

## Introduction

Since the discovery of human leucocyte antigen (HLA) typing and its powerful impact on hematopoietic allogeneic bone marrow and stem cell transplantation (1), there has been much progress made with regards to the understanding between HLA antigen and HLA antibody including its role in successful graft function and prevention of graft versus host disease (GvHD) (2). The identification of donor specific HLA antibody (DSA) has been shown to predict graft failure (3, 4), making this knowledge more relevant in today’s age whereby there is increasing use of mismatched or haploidentical allogeneic hematopoietic stem cell transplantation. Various techniques have been established and constitute the desensitization protocols used prior to transplantation. These incorporate plasma exchange, rituximab, intravenous immunoglobulin and use of blood products to reduce or clear DSA (3). These strategies target a few pathways including depleting B cells, altering plasma cells, removing as well as modifying antibodies. We present a case whereby use of the anti-CD38 monoclonal antibody daratumumab (5) was able to successfully reduce DSA via its action of the cell surface glycoprotein highly expressed on plasma cells in a multiparous patient with early T-cell precursor acute lymphoblastic leukemia (ETP-ALL).

## Case description

A 56 year old non-Caucasian female who was diagnosed with ETP-ALL developed complications of severe neutropenic sepsis after induction chemotherapy with fludarabine, cytarabine, granulocyte-colony stimulating factor and idarubicin (FLAG-Ida). She developed multiorgan dysfunction (MOD) and required care in the intensive care unit (ICU) for 11 days in view of respiratory failure, hemodynamic instability and acute kidney injury. Bone marrow assessment done on day 28 of treatment demonstrated measurable residual disease (MRD) negative status via flow cytometry. However, due to the infective complications sustained, she was severely deconditioned with critical illness myopathy and was bedbound with residual MOD.

### Consolidation

As she achieved a MRD negative status whilst being physically and functionally impaired, we opted to explore a non-chemotherapy form of consolidation in order to 1) keep her in remission, 2) minimize toxicities to allow for organ function and functional performance status to improve. She was consolidated with oral venetoclax x 28 days and 1-weekly subcutaneous daratumumab (6, 7). The following pre-medications were given before daratumumab administration: PO chlorpheniramine 4mg, PO paracetamol 1g, PO montelukast 10mg, PO famotidine 20mg. After 28 days, venetoclax was stopped because of worsening cytopenia. She also had a brief 2-week interruption of her daratumumab after developing cutaneous cytomegalovirus (CMV) infection for which she responded well to CMV-directed treatment. She was then maintained on weekly outpatient daratumumab and monthly intrathecal chemotherapy. During this time, she underwent aggressive rehabilitation to optimize her physical status whilst an allogenic stem cell transplant was being arranged.

### Pre-transplant donor selection

Donor availability was scarce as she had no healthy matched sibling donor nor matched unrelated donor options. Hence her three children were screened as potential donors for a haploidentical transplant. Unfortunately, she had strong DSAs against her children with mean fluorescence intensity (MFI) values of greater than 5000. The HLA antibody identification was done via solid phase immunoassay (SPI) based on the Luminex platform.

As she was on daratumumab for maintenance of her ETP-ALL, we opted to monitor her DSA to see if it would be reduced over time with daratumumab. At 10 weeks post daratumumab after receiving cumulatively 7 doses of daratumumab, a repeat DSA demonstrated significant reduction of her DSA to her daughters; whereby there was an 84% reduction of her DSA to A33:03 and a 36% reduction of her DSA towards B35:01. We decided to proceed with allogenic stem cell transplant with her haploidentical daughter as the donor with pre-transplant desensitization protocol.

### Transplant

**Figure 1:**
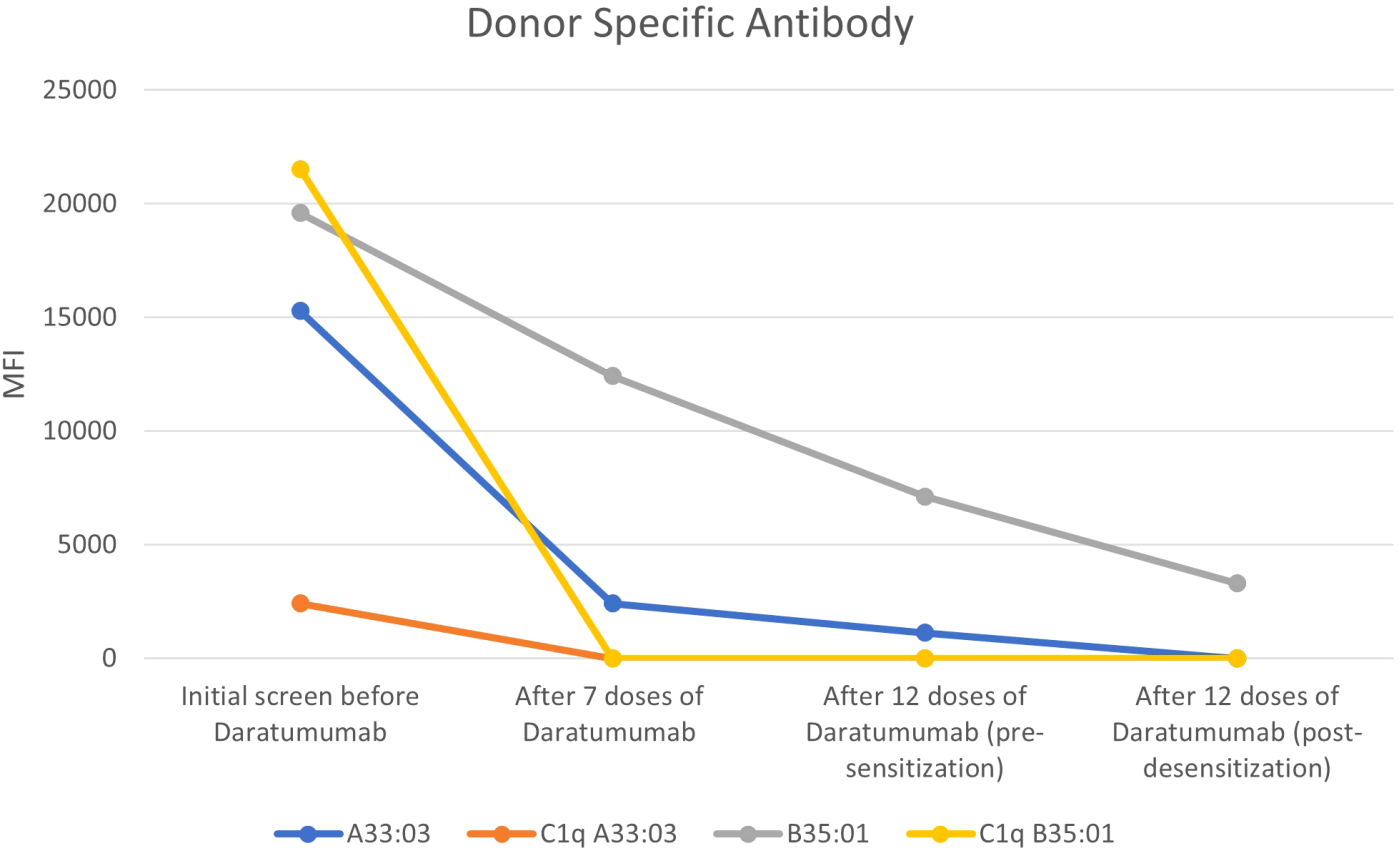
DSA trend done at initial screen, after daratumumab, pre- and post-desensitization.

She was conditioned with reduced intensity conditioning with thiotepa, fludarabine and treosulfan. Peripheral blood stem cell was given at a cell dose of 5.60 million CD34/kg. Post-transplant cyclophosphamide was chosen as GvHD prophylaxis in addition to myfortic and ciclosporin. Her post-desensitization MFI to A33:03 had disappeared and her MFI to B35:01 had significantly reduced to 3304. DSA trend found in **Table 1** and **Figure 1**.

**Table 1:** Summary of DSA trend done at initial screen, after daratumumab, pre- and post-desensitization.

DSA MFIMismatched HLA	Initial screen before daratumumab	After 7 doses of daratumumab	After 12 doses of daratumumab (pre-desensitization)	After 12 doses of daratumumab (post-desensitization)
A*33:03	15289	2418	1130	<1000
C1q A*33:03	2418	<1000	<1000	<1000
B*35:01	19601	12429	7117	3304
C1q B*35:01	21537	<1000	<1000	<1000

**Figure 2:**
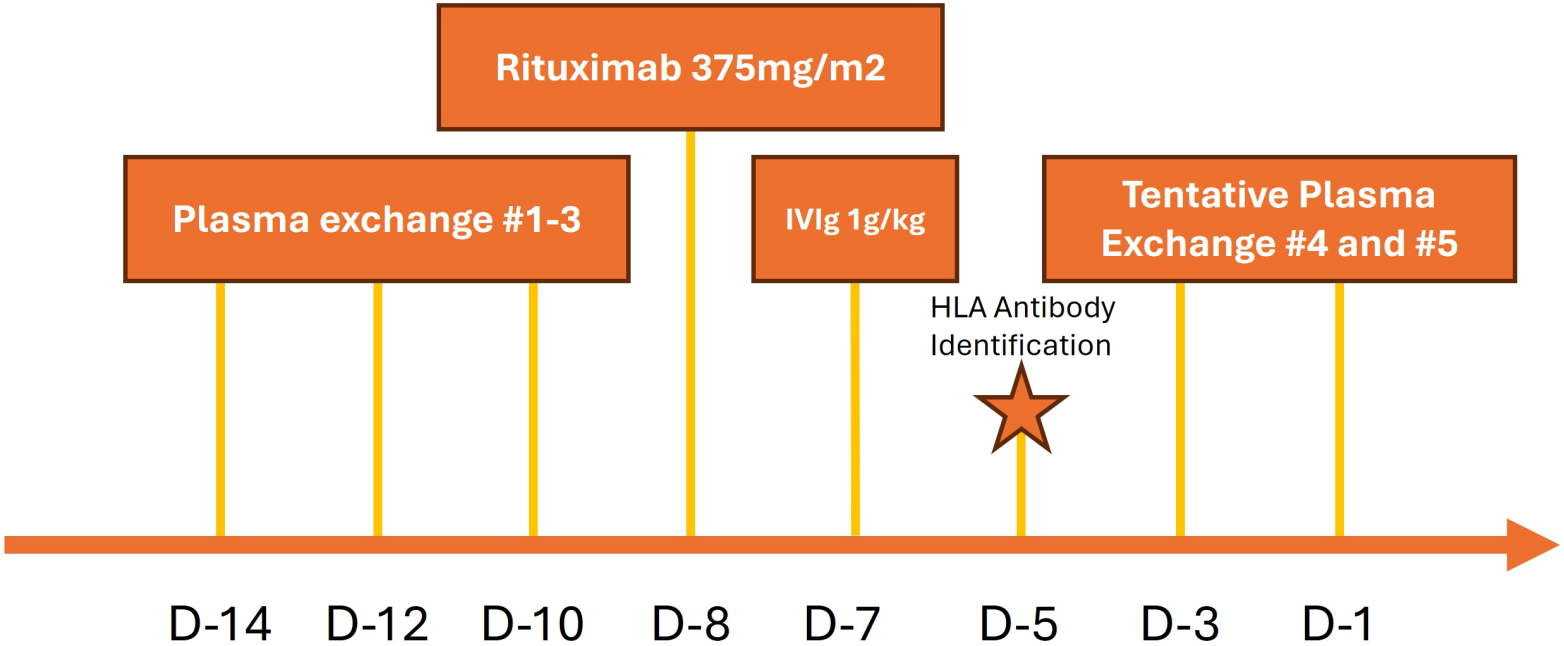
Desensitization protocol.

**Figure 2** depicts the desensitization protocol as per the standard operating procedures of the treating institution.

A chimerism analysis done 14 days post transplantation (D + 14) demonstrated 95-100% chimerism. She had neutrophil engraftment at D + 17 and platelet engraftment at D + 20. At D + 28, interval bone marrow assessment demonstrated no evidence of disease with negative MRD and chimerism analysis was 100%.

## Discussion

Early T-cell precursor (ETP) acute lymphoblastic leukemia (ALL) is a high-risk disease subtype of T-cell ALL with unique immunophenotypic and genomic features. It is characterized by the presence of CD7, absence of CD1a and CD8 and expression of one or more myeloid/stem cell markers (8, 9). Despite advancements in therapeutic strategies in ALL, ETP-ALL has been shown to have poor early treatment response to chemotherapy as well as significantly poorer long-term outcomes as compared to non-ETP-ALL patients (10, 11). In the search for identifying novel and effective treatment strategies in ETP-ALL, immunotherapy agents targeting its underlying molecular mechanisms have been sought (12–14). One such example is with the use of anti-CD38 monoclonal antibodies daratumumab and this has been supported by pre-clinical studies suggesting that CD38 is robustly expressed in T-ALL blasts thereby making it a potential effective target for daratumumab (15–17).

Along this same line, the use of anti-CD38 monoclonal antibodies have also been the agent of choice for tackling the important issue of DSA in transplantation (18, 19). The anti-CD38 monoclonal antibody exerts its therapeutic effect by targeting CD38, a cell surface glycoprotein highly expressed on plasma cells, thereby depleting antibody-producing plasma cells. Whilst there have been more experimental as well as clinical studies in evaluating daratumumab in kidney transplantation (20–23), its use has been shown to be effective in reducing DSA especially in cases whereby the standard of care desensitization protocols had been proven to be ineffective in reducing the MFI to less than moderate levels (24). Prior case reports in the use of daratumumab for desensitization of DSA were in patients with post essential thrombocythemia myelofibrosis, B-cell acute lymphoblastic leukemia, severe aplastic anemia and sickle cell disease (18, 24–26). Our case is the first reported patient with ETP-ALL to have shown the use of daratumumab in both controlling the underlying hematological disease process as well as reducing DSA levels effectively. In conclusion, daratumumab may be considered as a second line or salvage option for desensitization of DSA prior to haploidentical stem cell transplantation especially in multiparous ethnic-minority patients whom we often grapple with the issues of lack of donor availability and multiple DSA.

### Patient perspective

At the time when daratumumab was used as consolidation therapy for ETP-ALL, patient and family were agreeable with an alternative novel therapy that provided her with an opportunity to undergo a period of pre-rehabilitation leading up to the allogeneic hematopoietic stem cell transplant. This was an important step as she had developed multiple treatment related complications during induction therapy rendering her significantly deconditioned and frail. Prompt clearance of DSA prior to transplant was an unexpected and additional benefit observed in the setting of limited donor options for which her daughter was eventually selected as a haploidentical donor. Her transplant admission, engraftment and immediate post transplant recovery was uneventful. Unfortunately, 4 months post transplant, patient succumbed to a life threatening infection and passed away. Her last disease reassessment prior to her deterioration showed that she was in complete remission with full donor chimerism.

## Data Availability

The original contributions presented in the study are included in the article/supplementary material. Further inquiries can be directed to the corresponding author.
